# Chitosan and Essential Oils Combined for Beef Meat Protection against the Oviposition of *Calliphora vomitoria*, Water Loss, Lipid Peroxidation, and Colour Changes

**DOI:** 10.3390/foods11243994

**Published:** 2022-12-09

**Authors:** Priscilla Farina, Roberta Ascrizzi, Stefano Bedini, Antonella Castagna, Guido Flamini, Monica Macaluso, Alessia Mannucci, Ylenia Pieracci, Annamaria Ranieri, Maria Calogera Sciampagna, Francesca Venturi, Barbara Conti

**Affiliations:** 1 Department of Agriculture, Food and Environment, University of Pisa, Via del Borghetto 80, 56126 Pisa, Italy; 2 Department of Pharmacy, University of Pisa, Via Bonanno 6, 56126 Pisa, Italy

**Keywords:** *Laurus nobilis*, *Piper nigrum*, chemical analysis, sensory analysis, colourimeter, blowflies, Calliphoridae, repellents, dehydration, thiobarbituric acid

## Abstract

Meat production has a higher economic and ecological impact than other commodities. The reduction in meat loss and waste throughout the whole supply chain is a demanding challenge. In recent years, the interest in the food-grade polysaccharide chitosan (CH) and essential oils (EOs) employed as allies in meat protection has increased. In this work, we selected five EOs obtained from plants traditionally used as spices, and after their chemical characterisation, a trained panel of expert sensorial analysts determined that, among them, *Laurus nobilis* (Lauraceae) and *Piper nigrum* (Piperaceae) EOs were the most suitable to season meat. Therefore, the effect of CH, the *L. nobilis* and *P. nigrum* EOs, and EOs-enriched CH solutions on meat was tested to assess how they deter the oviposition behaviour of the blowfly *Calliphora vomitoria* (Diptera: Calliphoridae) and against water loss, lipid peroxidation, and colour changes. All the applied treatments, compared to the control, did not accelerate meat dehydration but increased colour lightness, an attractive feature for consumers, and discouraged the blowfly’s oviposition. In particular, the *P. nigrum* EO-enriched CH was the most active in repelling *C. vomitoria* without negatively affecting the organoleptic qualities and shelf-life of meat.

## 1. Introduction

According to the Food and Agriculture Organization [1], global meat production in 2020 accounts for about 337.3 million tonnes, and around 4% of the worldwide food loss and waste is exactly represented by this commodity [2]. Even if this percentage is lower than that of cereals, fruits, and vegetables, meat production has a higher economic and ecological impact. Indeed, it leads to the highest greenhouse gas emissions among all foodstuffs [3]. In less developed countries, loss and waste are localised at the production and storage levels due to inadequate infrastructures and technologies [2]; in industrialised regions, such as Europe, they occur during distribution, as well as at the retail and home consumption stages [4]. 

Meat products have a relatively short shelf-life and require undisrupted cold temperatures along the transport network to avoid spoilage [5]. Lipids, pigments, proteins, and vitamin oxidation are critical processes that also negatively affect meat quality [6].

The meat supply chain is also threatened by the Diptera Calliphoridae flies, commonly known as blowflies. In slaughterhouses, industries, and stores, if hygienic conditions are not optimal, blowflies target meat for their oviposition. The resulting maggots feed on the infested products causing their rotting and spoilage [7,8]. Moreover, adult blowflies can act as mechanical vectors of pathogenic bacteria and protozoa [9,10] as they come into contact with septic matters while promiscuously landing on different substrates and surfaces. According to the World Health Organization [11], the consumption of food contaminated by microorganisms leads to 600 million cases of foodborne diseases worldwide every year. 

In order to prevent and reduce meat loss and waste, it is necessary to adopt innovative and sustainable strategies for its protection at every stage, from handling to processing and storage. A promising natural and renewable substance is chitosan (CH), a food-grade polysaccharide composed of β-(1,4)-linked-d-glucosamine and N-acetyl-d-glucosamine units. CH is produced by deacetylation from chitin, which is the second most abundant existing polysaccharide, being the main constituent of fungi cell walls and arthropods’ exoskeletons [12]. CH already finds numerous applications in medicine, agriculture, food preservation, and the packaging industry [12].

Essential oils (EOs) have been proposed as eco-friendly repellents protecting foodstuffs from the attack of several insect pests, Calliphoridae flies included [7,8,13,14,15,16]. Many EOs, besides their pharmaceutical application, are safe for human consumption, and their use as flavourings is indicated in Regulation (EC) No. 1334/2008 [17].

Thus, this work aimed to select an EO with a suitable olfactory profile to be added to CH and to evaluate the EOs-enriched CH effectiveness in preserving the meat against the oviposition of the blowfly *Calliphora vomitoria* L. (Diptera: Calliphoridae), dehydration, lipid peroxidation, and colour changes.

## 2. Materials and Methods

### 2.1. Essential Oils Purchase and Chemical Characterisation

All the EOs used for the trials were purchased from commercial suppliers. The *A. sativum* EO was bought from Vis Medicatrix Naturae s.r.l. (Florence, Italy); *L. nobilis* from Fitomedical s.r.l. (Binasco, Italy); *S. rosmarinus* (=*R. officinalis*) from KOS Laboratorio di Erboristeria s.r.l. (Carmignano, Italy); *O. basilicum* methyl chavicol chemotype; and *P. nigrum* from Sigma-Aldrich (St. Louis, MO, USA).

The chemical characterisation was conducted at the Department of Pharmacy of the University of Pisa, Italy. For each EO, the whole procedure was repeated three times. EOs were diluted to 0.5% in HPLC-grade *n*-hexane and then injected into a GC–MS apparatus. Gas chromatography–electron impact mass spectrometry (GC–EIMS) analyses were performed with an Agilent 7890B gas chromatograph (Agilent Technologies Inc., Santa Clara, CA, USA) equipped with an Agilent HP-5MS (Agilent Technologies Inc.) capillary column (30 m × 0.25 mm; coating thickness 0.25 μm) and an Agilent 5977B single quadrupole mass detector (Agilent Technologies Inc.). 

The analytical conditions were as reported in Bedini et al. [8]: briefly, injector and transfer line temperatures 220 and 240 °C, respectively; oven temperature programmed to rise from 60 to 240 °C at 3 °C/min; helium as carrier gas at 1 mL/min; injection of 1 μL (0.5% HPLC grade *n*-hexane solution); split ratio 1:25. Acquisition parameters were as follows: full scan; scan range of 30–300 *m/z*; scan time of 1.0 s. The identification of the constituents was based on a comparison of the retention times with those of authentic samples, comparing their linear retention indices relative to the series of *n*-hydrocarbons. Computer matching was also used against commercial [18] and laboratory-developed mass spectra libraries built up from pure substances and components of commercial EOs of known composition and MS literature data [19].

### 2.2. Chitosan and Essential Oils-Enriched Chitosan Solutions

Highly viscous chitosan (CH) from crab shells, molecular weight ~50,000, CAS-No: 9012-76-4, was purchased from Sigma-Aldrich (St. Louis, MO, USA). For all the solutions, the protocol by Peng and Li [20] was followed, with minor changes. For the 0.5, 1.0, and 2.0% (*w*/*v*) plain CH solution, 0.5, 1.0, and 2.0 g of CH were, respectively, dispersed in 100 mL of demineralised water containing 1.0% (*v*/*v*) of glacial acetic acid (Carlo Erba Reagents s.r.l., Cornaredo, Italy). The solution was then stirred on a hot plate stirrer (new type, VELP Scientifica, Usmate, Italy) at 25 °C and 7× *g* for 2 h. For the EOs-enriched CH solutions, 0.5% (*v*/*v*) of vegetal glycerol (A.C.E.F. s.p.a., Fiorenzuola d’Arda, Italy), 0.6% (*v*/*v*) of Tween^®^ 80 (Sigma-Aldrich), and 0.1 or 1.0% (*v*/*v*) of the five selected EOs were added to the previously dissolved CH. The EOs concentration was adjusted based on the quantity of the solution employed in the different trials, as explained in Section 2.4 and Section 2.7. Successively, the EOs-enriched CH solutions were homogenised on a hot plate stirrer at 18 °C and 28× *g* for 4 min. Glycerol is a plasticiser that improves the CH mechanical properties, and Tween^®^ 80 is a surfactant used to ensure wettability [21]. The obtained solutions were stored at 4 °C for no longer than 7 days and heated to 18 °C before use. We prepared the solutions for the sensory analysis and colour assessment of meat during the pre-screening, oviposition deterrence trial with *C. vomitoria*, and meat preservation and quality analysis during storage. 

### 2.3. Selection and Training of Assessors 

The selection and training of assessors were performed according to the Department of Agriculture, Food and Environment (DAFE) of the University of Pisa internal procedure, which is based on a normalised technical procedure reported in the literature [22], with some modifications. 

All the potential new assessors have been involved in a multi-step training period arranged every year to select a sub-group of future panellists, characterised by the necessary motivation during the whole activity (attendance at more than 75% of training sessions), together with the minimum sensory skills required for food tasting and description (including visual, aroma, and taste attributes).

This multi-step general training is arranged over a period of three months as follows:

1. Theoretical introduction to the principles of human physiology of sight, smell, and taste.
2. Arrangement of preliminary training tests, mainly based on the utilisation of model standard solutions, to collect information about the tasting capacity of each assessor (i.e., sensory acuity, odour and flavour memory, term use and recall, scoring consistency).
3. As the discrimination relies as much on odour memory (that accumulates with experience) as on sensory acuity, ten tasting sessions were carried out in the morning, in a well-ventilated quiet room and in a relaxed atmosphere to evaluate different commercial foods. A sub-group of panellists (eleven people, three males and eight females, ranging from 26 to 65 years old) was selected, starting from the assessors already included in the official panel of the DAFE. All the assessors had previous experience in the food and EOs sensory descriptive analysis and were provided with a specifically developed sensory sheet consisting of a non-structured, parametric, and descriptive scoring chart. Furthermore, all the assessors were also asked to provide a list of some specific olfactory descriptors freely chosen to describe the olfactory profiles of the different samples tested.

### 2.4. Meat, Chitosan, Essential Oils, and Essential Oils-Enriched Chitosan Solutions Sensory Analysis

Samples for sensory analysis were prepared as described below:

1. Raw beef mince with 9% of fat (3 g + 600 µL of water) in a cubic embedding mould (2.1 cm side);
2. 100.0 µL of 1.0% *A. sativum*, *O. basilicum*, *L. nobilis*, *P. nigrum*, or *S. rosmarinus* EOs in ethanol (EtOH) (corresponding to 1.0 µL EO sample^−1^) on a fragrance tester strip;
3. 1.0 mL of 2.0% plain CH solution on a glass Petri dish (5.0 cm diameter);
4. 1.0 mL of 2.0% CH solution containing 0.1% of one of the five EOs (corresponding to 1.0 µL EO sample^−1^) on the glass Petri dish;
5. Raw beef mince (3 g + 600 µL of water) with 100 µL of 1.0% EtOH solutions of one of the five EOs (corresponding to 1.0 µL EO sample^−1^) in the embedding mould;
6. Raw beef mince (3 g + 600 µL of water) with 1.0 mL of 2.0% plain CH solution in the embedding mould;
7. Raw beef mince (3 g + 600 µL of water) with 1.0 mL of 2.0% CH solution containing 0.1% of one of the five EOs (corresponding to 1.0 µL EO sample^−1^) in the embedding mould.

In order to obtain the same quantity of EO in the pertinent samples (2, 4, 5, and 7), the used concentration of EO (0.1 or 1.0%) was adjusted based on the quantity of the employed solution (100.0 µL or 1.0 mL).

**Figure 1:**
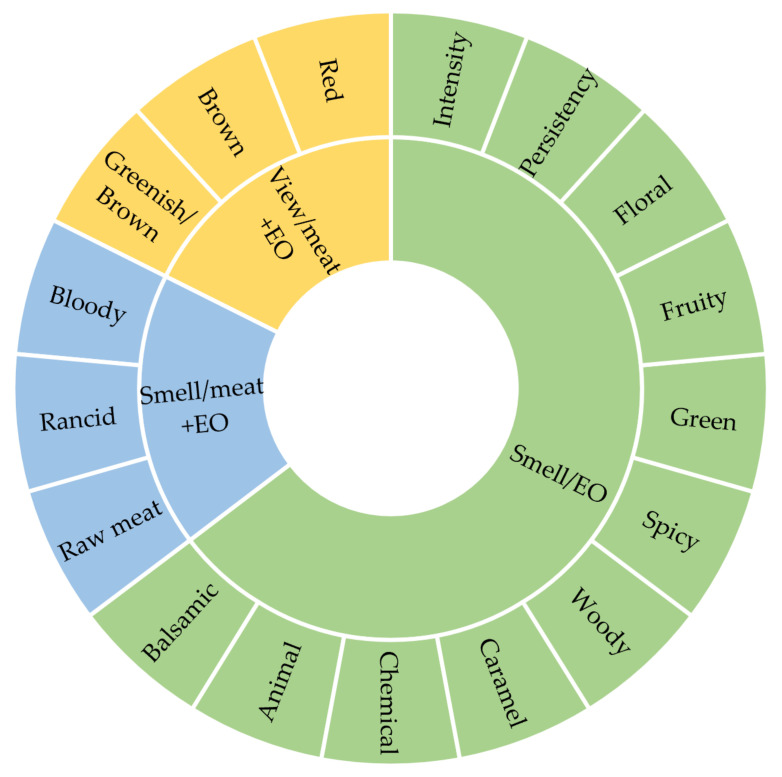
Sensory wheel for the essential oils (EOs) and meat + EOs evaluation (view and smell).

The trained panel of the DAFE of the University of Pisa evaluated the smell profiles of all the samples following the sensory wheel reported in Figure 1. 

### 2.5. Determination of Colour Coordinates (L*, a*, b*)

For the determination of the chromatic characteristics of raw beef mince, an Eoptis CLM-196 colourimeter (Eoptis S.r.l., Trento, Italy) was used. The instrument interfaces through the USB port to a PC with a Microsoft Windows operating system. The acquired colour values are expressed using the native CIE (Commission Internationale de l’Éclairage) coordinates L*, a*, and b* (CIELAB), according to the official method OIV-MA-AS2-11. L* defines the colour lightness (with L* = 0 black and L* = 100 white); a* is the position between red and green (−a* = green and +a* = red); b* is the position between yellow and blue (−b* = blue and +b* = yellow) [23]. The identification of colours in the CIELAB space can also be performed using the so-called cylindrical coordinates: h* and C*. h* defines the psychometric hue, while C* defines the psychometric chroma; they are related, respectively, to the perceptual terms of hue and saturation [23]. 

The Chroma value C* was calculated by the relation:

(1) $$C^{*}=\sqrt{a^{*}{}^{2}+b^{*}{}^{2}}$$

The colour difference among samples was expressed as $\Delta E_{\mathrm{ab}}^{*}$:

(2) $$\Delta E_{\mathrm{ab}}^{*}=\sqrt{\Delta L^{*}{}^{2}+\Delta a^{*}{}^{2}+\Delta b^{*}{}^{2}}$$

### 2.6. Calliphora vomitoria Rearing

**Figure 2:**
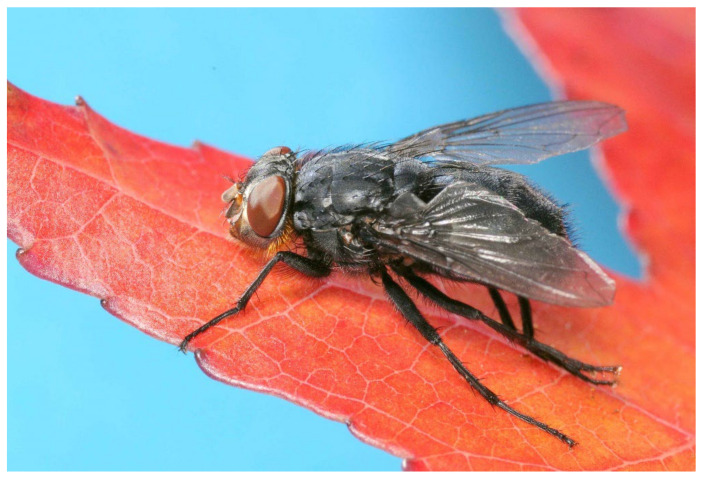
The blowfly *Calliphora vomitoria* L. (Diptera: Calliphoridae).

The whole rearing procedure was carried out according to Farina et al. [16], with minor changes. *C. vomitoria* mature larvae were purchased from the commercial supplier Altomare (Vittoria Apuana, Italy) and reared in a plastic box (27 × 21 × 12 cm) with a netted lid for ventilation. Larvae were fed with raw beef mince and kept under laboratory conditions (temperature 23 °C, RH 60–70%, natural photoperiod) until pupation. Adult blowflies (Figure 2), after the species identification [24], were reared in a 75.0 × 75.0 × 115.0 cm polyester and knitted mesh tent (BugDorm-2400 Insect Rearing Tent, MegaView Science Co., Ltd., Taichung, Taiwan) under the same laboratory conditions. Adults were fed a solid diet (sucrose and yeast extract 4:1) and water ad libitum. Yeast was proven to be necessary to provide the proteins needed to stimulate oviposition in Diptera [25].

### 2.7. Calliphora vomitoria Oviposition Deterrence Trial

For the oviposition deterrence assays, adults of *C. vomitoria* were moved into 47.50 × 47.50 × 93.0 cm nylon and knitted mesh cages (BugDorm-4M4590DH, MegaView Science Co., Ltd., Taichung, Taiwan). Each cage contained one hundred and fifty unsexed blowflies (sex ratio 1:1), 10–20 days old, fed a solid diet (sucrose and yeast extract 4:1) and water ad libitum. Cages were also furnished with a beaker covered by cotton gauze containing 500 mL of water to maintain humidity and were kept under fluorescent lamps (14,000 lux) to provide even lighting during the whole duration of the trials, at 23 °C and RH 60–70%. The methodology was adapted from Bedini et al. [13,14,15] with minor changes. 

**Figure 3:**
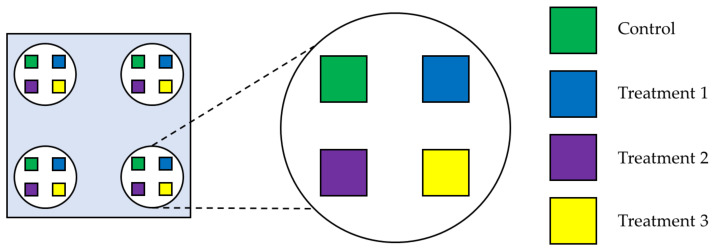
Schematic representation of how the moulds containing the beef meat were arranged in the oviposition deterrence trial. The grey square represents the cage seen from above; the green, blue, purple, and yellow squares represent the moulds containing the meat samples (one control and three different treatments); the white circles represent the supports to which the moulds are glued.

Firstly, the protection against *C. vomitoria* oviposition given by the *L. nobilis* and *P. nigrum* EOs was evaluated. In each cage, a total of sixteen cubic embedding moulds (2.1 cm side) were positioned; they were filled with 5 g of raw beef mince with 9% of fat and added with 1.0 mL of water to avoid dehydration. The meat surface was flattened and treated with 100 µL of 0.0 (control, CTR), 0.5, 1.0, and 2.0% EtOH solutions of one of the two EOs (corresponding to 0.0, 0.5, 1.0, and 2.0 µL EO sample^−1^). Four moulds, each one containing one of the different EO concentrations, were positioned in correspondence with the four inner corners of the cage, at about 5 cm from the edges, as schematised in Figure 3. The bases of the moulds were glued with double-sided tape to a circular lid (10.0 cm diameter) to avoid overturning. The test lasted 24 h, during which the female blowflies were free to lay their eggs in the preferred sample.

Afterward, following the same protocol and scheme (Figure 3), the protection given to the meat samples by 1.0 mL of 0.5, 1.0, and 2.0% plain CH solutions were tested, compared with an untreated meat CTR. 

By taking into consideration the previously obtained results, the need to use the lowest concentration possible of EOs to propose an economically advantageous treatment, and the ease of application of the treatments based on their fluidity, the 1.0% EtOH EO and 1.0% plain CH solutions were selected. Therefore, the protective effect of 100 µL of the 1.0% EtOH solution of *L. nobilis* or *P. nigrum* EOs (corresponding to 1.0 µL EO sample^−1^) was compared to 1.0 mL of the 1.0% CH solution, 1.0 mL of 1.0% CH solution containing 0.1% of one of the two EOs (corresponding to 1.0 µL EO sample^−1^), and an untreated CTR (Figure 3). In order to obtain the same quantity of EO in the pertinent samples, the used concentration of EO (0.1 or 1.0%) was adjusted based on the quantity of solution employed (100.0 µL or 1.0 mL).

All the experiments were replicated three times, applying the same methodology. The laid eggs were counted 24 h from the beginning of the assays, using the piece counter function of an analytical balance (KERN ABS-N, Kern & Sohn, Balingen, Germany). The protection of the different treatments against *C. vomitoria* was assessed as the percentage of oviposition according to the following formula: NT ÷ NCG × 100, where NT is the number of eggs laid on the specific treatment, and NCG is the total number of eggs laid in the cage.

### 2.8. Meat Preservation and Quality Analysis

The effect of the CH edible coatings, with or without the EOs enrichment, was tested on the shelf-life of raw beef mince with 9% of fat. All the CH solutions were prepared as reported in Section 2.2. Meat patties (10.43 ± 0.07 g weight, 3.5 cm diameter) were manually made and treated with 1.0 mL of the 1.0% plain CH solution, 0.1% of the *L. nobilis* or *P. nigrum* EOs, and 1.0% CH solution enriched with 0.1% of EO (*L. nobilis* or *P. nigrum*). The 1.0 mL treatments were applied to the patties by spraying them, and the coated samples were stored at 5 °C in plastic Petri dishes (8.5 cm diameter), simulating home storage conditions. Treated beef patties were compared to control (CTR) and untreated patties, and the analysis was performed after the coatings solidified on the beef surface (day 0).

The weight loss percentage and colour determination (L*, a*, and b*—CIELAB) were assessed on days 0, 4, and 7 (*n* = 6 for each group and time). Meat patties were further analysed to evaluate the lipid peroxidation status by measuring the concentration of the thiobarbituric acid reactive substances (TBARS) at each time point, using a pro-UV–vis spectrophotometer (Amersham Biosciences Ltd., Amersham, UK). In detail, samples were homogenised in 5% trichloroacetic acid (TCA, 1 g:10 mL *w*/*v*) and centrifuged at 10,000× *g* for 20 min at 4 °C; after that, the supernatant was collected. The extract (200 μL) was added to 1.0 mL of either TBA (thiobarbituric acid) solution (15% TCA and 0.01% butylated hydroxytoluene) or + TBA solution (15% TCA, 0.375% TBA, 0.01% butylated hydroxytoluene). Samples were then shaken and boiled at 100 °C within a block heater for 20 min. Before analysis, samples were let to cool down in an ice bath, and the absorbance was then read at 532, 440 and 600 nm. The results were expressed as nmol of malondialdehyde (MDA) equivalent g−^1^ FW [26,27].

### 2.9. Data Analysis

The results of the sensory analysis were processed by the Big Sensory Soft 2.0 software (version 2018, Centro Studi Assaggiatori, Brescia, Italy). Sensory data were analysed by two-way ANOVA with panellists and samples taken as main factors [28]. 

Differences in the oviposition of *C. vomitoria* among treatments were assessed by one-way ANOVA, with the percentage of laid eggs as the dependent variable and the treatment as the main factor. Means were separated by Tukey HSD post hoc test. Oviposition percentage data were transformed into arcsine values before statistical analysis. Data were processed by SPSS 22.0 software (SPSS Inc., Chicago, IL, USA). 

One-way ANOVA and Tukey HSD post hoc test was also applied to assess weight loss, lipid peroxidation, and colour changes during the storage of raw beef patties, with the treatment as the main factor. In the case of colour determination, the effect of the time of storage was also checked for each treatment.

## 3. Results

### 3.1. Pre-Screening of the Essential Oils to Be Used for Meat Storage

#### 3.1.1. Chemical Composition of the Essential Oils

The complete composition of all the analysed EOs is reported in Table 1.

In the *A. sativum* EO, 19 compounds (86.1% of the total composition) were detected, all belonging to the non-terpene sulphur derivatives chemical class, of which the most abundant were diallyl tetrasulphide (27.3%) and *di*-2-propenyl trisulfide 18.3%.

Sixty-two compounds were identified in the *L. nobilis* EO (99.2% of the total composition). Oxygenated monoterpenes constituted the most represented chemical class, among which 1,8-cineole (28.1%) and α-terpinyl acetate (17.5%) were the most abundant. Other quantitatively relevant chemical groups were monoterpene hydrocarbons (15.6%) and phenylpropanoids (8.4%). Among the former, sabinene (4.7%) and α-pinene (3.6%) were the most represented, while the latter was mainly composed of methyl eugenol (7.3%) and eugenol (3.4%).

The *O. basilicum* EO was characterised by 31 compounds (99.6% of the total composition), of which 80.9% were phenylpropanoids, chiefly represented by methyl chavicol (76.3%).

In the *P. nigrum* EO, 39 compounds were detected (100% of the total composition), of which over 60% were represented by sesquiterpene hydrocarbons. Among them, β-caryophyllene reached up to 45.7%. Monoterpene hydrocarbons followed (31.3%), with limonene as the most abundant (8.0%).

Twenty-nine compounds were identified in the *S. rosmarinus* EO (100% of the total composition). Over 60% were represented by oxygenated monoterpenes, of which 1,8-cineole accounted for up to 41.1%.

#### 3.1.2. Meat, Chitosan, Essential Oils, and Essential Oils-Enriched Chitosan Solutions Sensory Profiles

**Figure 4:**
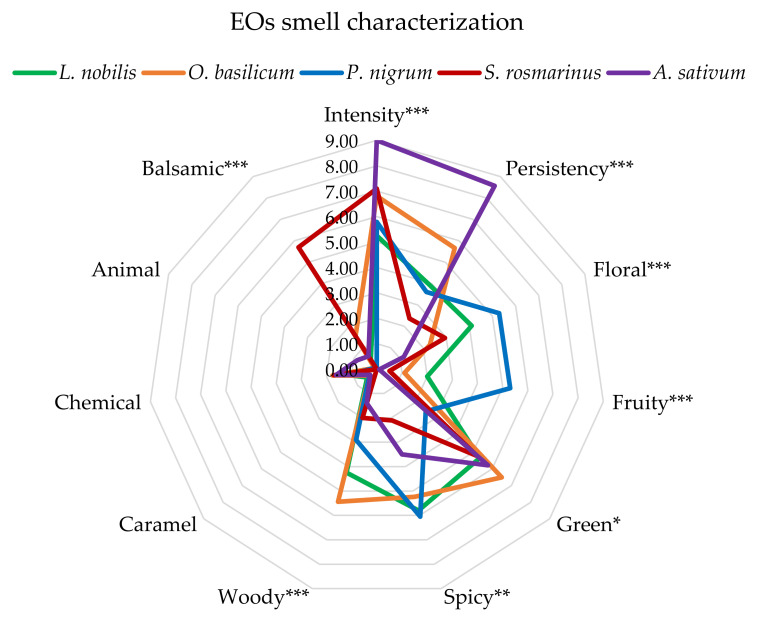
All the smell descriptors attributed by the trained panel to the *Allium sativum*, *Laurus nobilis*, *Ocimum basilicum*, *Piper nigrum,* and *Salvia rosmarinus* essential oil (EO) on a 0–9 scale. Significance level. *** = *p* < 0.001; ** = *p* < 0.01; * = *p* < 0.05;   = not significant (*p* > 0.05).

The EOs selected for the treatment of meat, chosen among the spices traditionally used to season meat dishes [30], were *A. sativum*, *L. nobilis*, *O. basilicum*, *P. nigrum*, and *S. rosmarinus*. Figure 4 shows the overall descriptors used to define their profiles before their utilisation on meat samples. In order to complete the analysis, panellists were asked to list some specific descriptors when necessary (Table 2). According to the compositions shown in Table 1, the best smell profiles were attributed to the *L. nobilis* and *P. nigrum* EOs, with high scores on the floral, fruity, and spicy descriptors (Table 2). On the contrary, given the presence of several compounds with aromatic sulphur notes (Table 1), the *A. sativum* EOs were characterised by a high number of unpleasant aromas (Table 2), together with the highest smell intensity and persistency (Figure 4).

**Figure 5:**
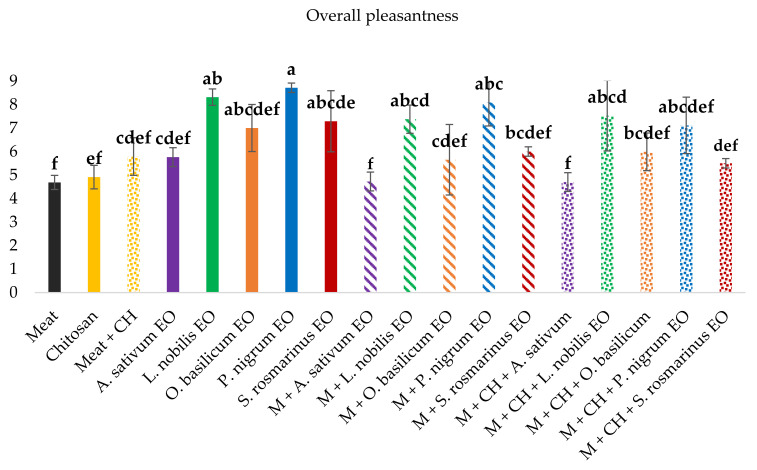
Overall pleasantness of all the samples evaluated by the trained panel on a 0–9 scale. Meat (M); chitosan (CH); *Allium sativum*, *Laurus nobilis*, *Ocimum basilicum*, *Piper nigrum*, and *Salvia rosmarinus* essential oil (EO). Different letters (a–f) indicate significant differences.

Figure 5 shows the overall pleasantness attributed to all the EOs EtOH solutions together with data related to the meat samples treated with the various EOs, CH, and EOs enriched CH solutions. Among the selected five EOs, the *P. nigrum* showed the highest score for overall pleasantness, closely followed by *L. nobilis*, while the lowest score was attributed to the *A. sativum* EO. The latter was below the acceptability limit, generally fixed at 5 when 9 is the maximum score value. 

Without treatment, the smell of plain meat was described as rancid and cadaverine-like, while that of plain CH was described as acetic and acetone-like due to the use of glacial acetic acid for its preparation (Section 2.2). Nevertheless, the presence of CH reduced the smell intensity of the treated meat, thus reducing the off-flavour detection and improving the global pleasantness score.

Overall, the presence of CH did not significantly affect the sensorial profile of the meat treated with the EOs. When the meat was treated with the EOs or EOs-enriched CH, the best sensorial profiles were obtained with the *L. nobilis* EOs and *P. nigrum*, while the worst ones were associated with the *A. sativum* EO. Furthermore, meat samples treated with the *O. basilicum* and *S. rosmarinus* EOs were close to the limit of acceptability, regardless of the presence of CH.

#### 3.1.3. Colourimetric Determination

Soon after treatment, the visual appearance of the meat samples treated with all the combinations of EOs and EOs-enriched CH solutions was deeply affected by the treatment. Table 3 shows that, when meat was treated with the EOs-enriched CH solutions, the colour was generally less vivid. When using the *A. sativum* EO, the shade changed from red/brown to yellow/brown or greenish/brown. 

### 3.2. Calliphora vomitoria Oviposition Deterrence Activity

The oviposition deterrence assays indicated that both the EOs and plain CH could strongly affect the oviposition behaviour of *C. vomitoria* females. By using the plain CH solutions alone, the *C. vomitoria* oviposition was reduced up to eleven times (*F*_3,11_ = 18.887, *p* = 0.001), but with no significant differences among CH concentrations (0.5, 1.0, and 2.0%) (Tukey HSD, *p* > 0.05). 

**Figure 6:**
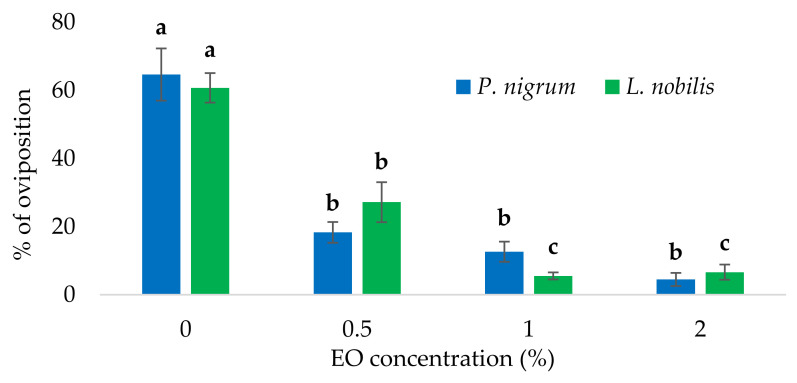
Protective effect of different concentrations of the *Laurus nobilis* and *Piper nigrum* essential oils (EOs) against the oviposition of the blowfly *Calliphora vomitoria* on beef meat. For each EO, different letters (a–c) indicate significant differences among concentrations (Tukey’s HSD, *p* ≤ 0.05).

Similarly, a clear repellent effect was observed for the EO-treated samples, with significant differences both for the *P. nigrum* (*F*_3,11_ = 36.332, *p* < 0.001) and *L. nobilis* EOs (*F*_3,11_ = 45.011, *p* < 0.001). However, while no significant differences were detected among different concentrations of the *P. nigrum* EO (0.5, 1.0, and 2.0% in EtOH) (Tukey HSD, *p* > 0.05), the effect of the *L. nobilis* EO was dose-dependent with significant differences among the concentrations. In detail, for the *L. nobilis* EO, the post hoc test indicated a significant difference between the 0.5 and 1.0% EO concentrations (Figure 6). 

**Figure 7:**
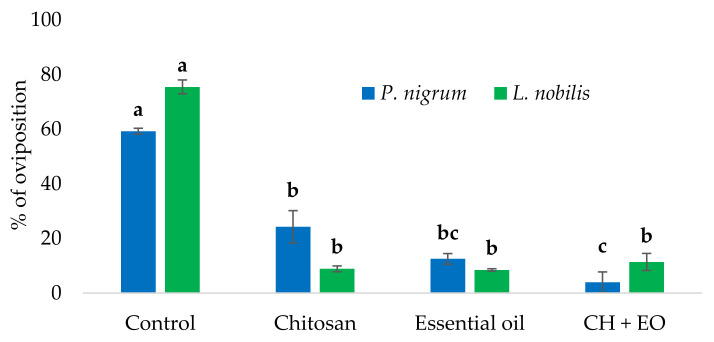
Protective effect of chitosan (CH), the *Laurus nobilis* and *Piper nigrum* essential oils (EOs), and EOs-enriched CH solutions against the oviposition of the blowfly *Calliphora vomitoria* on beef meat. For each EO, different letters (a–c) indicate significant differences among treatments with the same EO (Tukey’s HSD, *p* ≤ 0.05).

As previously explained in the Materials and Methods section (Section 2.7), we decided to use the treatments with 1.0% CH, 1.0% EOs, and 1.0% CH solution containing 0.1% of the EOs to be compared. In this case, the ANOVA showed significant differences among the treatments both for the *P. nigrum* (*F*_3,11_ = 43.676, *p* < 0.001) and *L. nobilis* (*F*_3,11_ = 248.649, *p* < 0.001) EOs. In detail, the post hoc test indicated that among the *P. nigrum* treated samples, the most effective treatment was the EO-enriched CH solution, whose effect was significantly stronger than that of the plain CH and CTR. On the contrary, among the *L. nobilis* treated samples, significant differences were shown only among the CH, EO, and the EO-enriched CH solution with the EO and the CTR (Figure 7).

### 3.3. Meat Characterisation during Storage

Given the results above discussed the sensorial characterisation of the proposed treatments and the protection they gave against the *C. vomitoria* oviposition, we also evaluated meat preservation for 7 days by treating the samples with the *L. nobilis* or *P. nigrum* EOs, CH, and the corresponding EOs-enriched CH solutions. 

#### 3.3.1. Weight Loss

**Figure 8:**
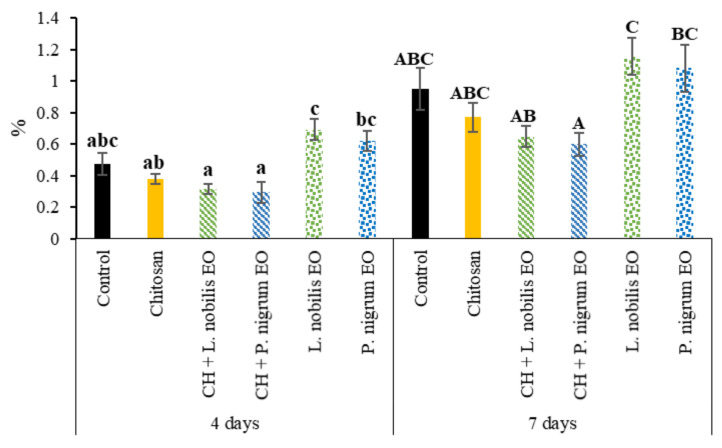
Weight loss (%) of beef patties during 7 days of cold storage subjected to different treatments. Chitosan (CH); *Laurus nobilis* or *Piper nigrum* essential oil (EO); EOs-enriched CH solutions (CH + *L. nobilis* EO; CH + *P. nigrum* EO). Data are expressed as mean ± standard error. For each day, different letters indicate differences according to Tukey HSD (*p* ≤ 0.05). Lower case letters (a–c) were used for 4 days of storage; upper case letters (A–C) were used for 7 days of storage.

Weight loss (%) was calculated in comparison to the initial weight of each sample (day 0). Significant changes in the weight loss percentage (Figure 8) were observed after 4 (*F*_5,30_ = 8.103; *p* < 0.0001) and 7 days (*F*_5,30_ = 4.1342; *p* < 0.01). All the treatments showed a similar trend in comparison with the CTR samples. However, after 4 days, the *L*. *nobilis* EO significantly differed from the CH treatments, both plain and enriched. Moreover, after both 4 and 7 days, the enriched CH solutions performed better than the respective EOs alone. 

#### 3.3.2. Colour Modifications

During storage, the colour was assessed by measuring the L*, a*, and b* parameters according to the CIELAB system (Table 4). The difference among treatments was compared to verify how the application of an edible coating may influence the attractiveness compared to the CTR beef patties. 

The lightness index L* was affected by the treatments at day 0 (*F*_5,30_ = 5.522; *p* = 0.001) and after 7 days (*F*_5,30_ = 7.111; *p* < 0.001). It is interesting to note that, at the beginning of the storage, samples coated with the CH enriched with the *L. nobilis* (+10.6%) and *P. nigrum* (+9.6%) EOs but also with plain CH (+10.5%) displayed a higher lightness compared to the CTR group. After 7 days, the enriched CH solutions (+10.5 % and +9.0% for *L. nobilis* and *P. nigrum*, respectively) and plain CH (+9.2%) still conferred higher lightness values to meat if compared to CTR. 

The a* coordinate was significantly affected by the treatments after 4 (*F*_5,30_ = 2.874; *p* < 0.050) and 7 days (*F*_5,30_ = 4.246; *p* < 0.010). In the first case, the only significant difference was found between the CTR and CH enriched with the *L. nobilis* EO, with the latter having a lower value of about −11%, while the other treatments had similar values to the CTR. After 7 days, all the treatments showed a lower a* compared to the CTR (−10.2%, −13.4%, −11.6%, −11.5% for CH, CH+*L. nobilis* EO, *L. nobilis* EO, and *P. nigrum* EO, respectively), except for the CH enriched with the *P. nigrum* EO, which was similar to the CTR.

The b* coordinate was significantly affected by the treatments applied only at day 0 (*F*_5,30_ = 4.778; *p* < 0.010). In particular, all the treatments showed no difference with the CTR group, but the CH-treated samples displayed a lower value if compared with the EOs treatments (−29.7% and −34.6%, for the *L. nobilis* and *P. nigrum* EOs, respectively) and CH enriched with the *P. nigrum* EO (−33.2%).

Besides the evaluation of the differences induced by the different coatings, the changes in the colour indexes occurring during storage were checked for each treatment (Table 4). While the greater changes in all the parameters (L*, a*, and b*) utilised to measure the meat’s colour were already evident after the first 24 h, regardless of the treatment, some further indications can be highlighted and discussed even during the 7 days storage.

In particular, for the plain CH, CH enriched with the *L. nobilis* or *P. nigrum* EOs, and *L. nobilis* EO, no changes for any of the coordinates investigated were found during the observation time. Both the CTR and CH samples showed a significant increase in a* after 4 and 7 days. Specifically, in CTR samples, a* was 14.4% and 20.7% higher (*F*_5,30_ = 11.419; *p* ≤ 0.001) after 4 and 7 days, respectively, as compared to the beginning of storage. The CH samples displayed a similar trend, with an increase in a* (*F*_5,30_ = 5.792; *p* < 0.050) of +13.1% at 4 days and +12.8% at 7 days compared to day 0. For samples treated with the *P. nigrum* EO only, L* underwent a little increase of +5.9 % (*F*_5,30_ = 4.290; *p* < 0.050) after 4 days of storage.

The total colour differences (ΔE_ab_) compared to the initial values (0 days) were calculated at 4 and 7 days of storage for each group (Table 4). During the whole observation period, CTR samples displayed the highest colour change, regardless of the preserving solution adopted. 

Moreover, when the total colour differences (ΔE_ab_) were calculated among samples on each day of storage (Table 5a–c), the higher ΔE_ab_ values were detected when chitosan was added to the meat, regardless of the storing time considered. 

#### 3.3.3. Lipid Peroxidation Index

**Figure 9:**
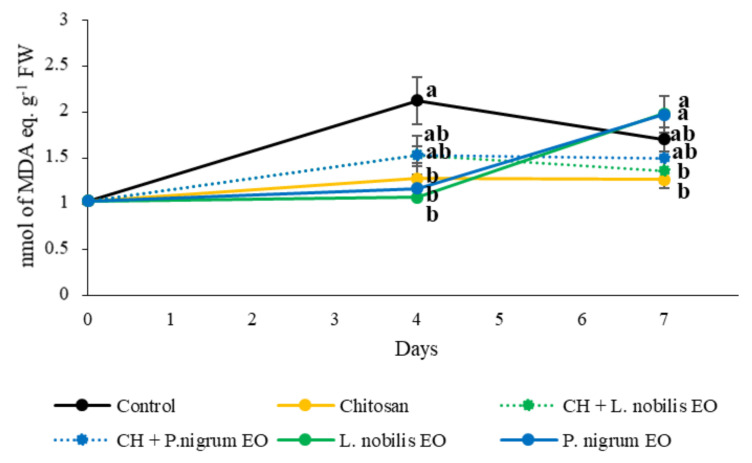
Lipid peroxidation index, expressed as nmol of malondialdehyde (MDA) equivalent g^−1^ FW, of beef patties for 7 days of cold storage subjected to different treatments. Chitosan (CH); *Laurus nobilis* or *Piper nigrum* essential oil (EO); EOs-enriched CH solutions (CH + *L. nobilis* EO; CH + *P. nigrum* EO). Data are expressed as mean ± standard error. Different letters (a,b) indicate significant differences among treatments (Tukey HSD, *p* ≤ 0.05) for each day.

The presence of secondary products of lipid oxidation (Figure 9) was evaluated at 0, 4, and 7 days of cold storage. After 4 days, the treatments applied on the beef patties’ surface caused some significant differences in this parameter (F_5,12_ = 6.030; *p* < 0.010). Indeed, CH and EOs, both the *L. nobilis* and *P. nigrum*, produced a reduction in the lipid peroxides concentration (−40%, −49%, and −44%, respectively) when compared to the CTR group, while the EOs-enriched CH had similar concentrations to the CTR and the other treatments.

The lipid peroxidation index was also significantly affected by the treatments at the end of the storage (*F*_5,12_ = 6.718; *p* < 0.010). In particular, no significant differences were found for all the treatments applied compared to the CTR group, even if a trend towards a lower lipid peroxidation index can be appreciated in the case of the CH and CH enriched with the *L. nobilis* EO; however, some differences emerged among the coating treatments. Specifically, the treatment with the two EOs alone, both the *L. nobilis* and *P. nigrum*, caused an increase in the TBARS concentration compared to the CH (+57% and +56%, respectively) and CH enriched with the *L. nobilis* EO (+46% and 45%, respectively). 

## 4. Discussion

Meat protection, preventing the loss and waste of this commodity with a particularly negative ecological impact, is a demanding challenge that must be addressed. In recent years, the interest in innovative and sustainable packaging able to improve the shelf-life of meat has increased. In this study, we assessed beef meat protection against oviposition by the blowfly *C. vomitoria* and its preservation using a CH edible coating mixed with two EOs (*L. nobilis* and *P. nigrum*) selected by expert sensorial analysts based on their suitability for meat.

The compositions of the EOs involved in this study were consistent with those reported in the pertinent literature. Sulphur-containing compounds, exhibited in different proportions, were the main components in the *A. sativum* EO. For example, 41 garlic accessions from Brazil showed wide ranges of diallyl disulphide (1.13–51.06%), diallyl trisulphide (27.86–57.06%), and diallyl tetrasulphide (0.55–21.35%) in their EOs compositions [31]. Torpol et al. [32] used two commercial garlic EOs containing, respectively, 31.67 and 27.19% of diallyl disulphide, 31.56 and 42.49% of diallyl trisulphide, and 13.48 and 9.92% of diallyl tetrasulphide.

The Moroccan *L. nobilis* EO used by Nafis et al. [33] revealed a composition similar to that reported in the present study, with 1,8-cineole (eucalyptol) as the main constituent (40.85%), followed by α-terpinyl acetate (12.64%) and methyl eugenol (8.72%). Two laurel EOs, one extracted from a Greek accession and one from a Georgian one, exhibited 1,8-cineole (30.8 and 29.2%, respectively) and α-terpinyl acetate (14.9 and 22.6%, respectively) as major components, as shown by the results of the present work. The EO from Greece also contained 8.0% of α-terpineol and 6.0% of terpinen-4-ol; the EO from Georgia was composed of 12.2% of sabinene and 8.1% of methyl eugenol [34]. 

The *O. basilicum* EO characterised in this paper was a methyl chavicol-chemotype (76.3%), as stated by the manufacturer. This chemotype was found in Turkey (city of Zonguldak), as reported by Telci et al. [35], and in Mississippi (United States), according to a study on 38 basil genotypes [36].

β-Caryophyllene is commonly reported as the main compound of *P. nigrum* EO: it accounted for up to 51.12% in a black pepper EO used by Andriana et al. [37], and a similar percentage (47.14–50.88%) was reported by Rmili et al. [38]. 

Similarly to the *S. rosmarinus,* EO analysed in the present work, Soulaimani et al. [39] indicated 1,8-cineole (31.13%), camphor (17.56%), and α-pinene (11.13%) as the main constituents in rosemary plants harvested in Morocco. The same components were also reported for other Moroccan plants grown at different altitudes (1,8-cineole 50.60–64.27%, camphor 1.77–14.12%, and α-pinene 6.61–9.02%) [40].

The five EOs proposed for meat preservation were initially selected based on their traditional use in meat seasoning [30]. Among them, the *P. nigrum* and *L. nobilis* EOs showed the best sensorial profile both in pure solution and in combination with meat, regardless of the presence of CH. On the contrary, the *A. sativum* EO showed the lowest overall pleasantness in all the conditions tested (EtOH solution, EO + meat, EO + CH + meat). With the only exception of the *A. sativum*, the addition of EOs significantly improved the sensorial profile of meat samples, regardless of the presence of CH. Furthermore, when the *A. sativum* EO was utilised, the colour of meat samples was also negatively affected.

The oviposition deterrence on *C. vomitoria* was already evaluated using several EOs extracted from culinary herbs. Complete meat protection was achieved using *A. sativum* EO at the concentration of 1.25 µL EO cm^−2^ [8] and *Artemisia dracunculus* L. (Asteraceae) EO at a substantially lower concentration of 0.05 µL EO cm^−2^ [13]. Three EOs from distinct *Origanum vulgare* L. (Lamiaceae) chemotypes offered different levels of protection. At the concentration of 0.32 µL EO cm^−2^, the thymol/γ-terpinene oregano chemotype EO avoided almost 90% of the oviposition, and the thymol/*p*-cymene and carvacrol chemotypes EOs more than 60% [15]. Similarly, our results show that, at the concentration of 0.48 µL EO cm^−2^, the *L. nobilis* and *P. nigrum* EOs exert protection of 89 and 93%, respectively.

To the best of our knowledge, none of the EOs used in this work has been used to control *C. vomitoria* before, but they were applied as repellents against other insect pests as well as insecticides. In a repellence assay on stored products pests, an *L. nobilis* EO at 78.63 nL EO/cm^2^ proved to be highly repellent (more than 80%) towards *Tribolium castaneum* (Herbst) (Coleoptera: Tenebrionidae) and *Liposcelis bostrychophila* Badonnel (Psocoptera: Liposcelididae) after 24 h of exposure [41]. A 3.0% *L. nobilis* EO formulated with olive oil protected for 52.3 min from *Culex pipiens molestus* Forskål (Diptera: Culicidae) bites [42]. Erler et al. [43] tested the repellence of an *L. nobilis* EO against *C. pipiens* female mosquitoes in a Y-tube olfactometer, reporting a more than 80% repellent effect with 10 µL of EO in an exposure time of 255 s.

Sticking to the repellence, Chaubey [44] found that a *P. nigrum* EO was 100% repellent on filter paper in Petri dishes starting from the concentration of 0.8% in acetone against *Sitophilus zeamais* (Motsch.) (Coleoptera: Curculionidae) and 97.5 ± 0.5% repellent from the concentration of 0.0125% in acetone against *Sitophilus oryzae* (L.) [45]. A different accession of a *P. nigrum* EO induced reduction in the oviposition and eggs hatching, a delay in the transformation of larvae into pupae, and a decrease in the final number of adults in *Callosobruchus chinensis* L. (Coleoptera: Bruchidae) [46].

CH finds various applications in insect pest control, both as a repellent and insecticide. Different CH concentrations (from 0.5 to 5%) were successfully used on paper and wood to inhibit the activity of the termites *Reticulitermes flavipes* (Kollar), *Reticulitermes virginicus* Banks, and *Coptotermes curvignathus* (Holmgren) (Isoptera: Rhinotermitidae) [47,48]. Moreover, several EOs have been added to CH matrixes to enhance their efficacy and persistence. *Melissa officinalis* L. (Lamiaceae) nanoencapsulated EO in CH (from 0.06 to 0.30 mL EO in 1.5% CH) showed antifeedant activity and toxicity by fumigation on *Tribolium castaneum* Herbst (Coleoptera: Tenebrionidae) [49]. *Cymbopogon* spp. (Poaceae) EO adsorbed on a CH and silica gel matrix successfully repelled adults of the mosquito *Aedes aegypti* L. (Diptera: Culicidae) for up to 4 h [50]. CH enriched with *Ferulago campestris* (Besser) Grecescu (Apiaceae) EO (from 10 to 25% EO in 2.0% CH) hindered the reproductive activity of *Acanthoscelides obtectus* (Say) (Coleoptera: Bruchidae) females on the common bean *Phaseolus vulgaris* L. (Fabaceae) [51].

Concerning meat dehydration during the 7 days of storage at cold temperatures, all the treatments had no effects if compared with the CTR group at any time point. However, EOs alone generally caused a higher loss compared to the CH enriched with EOs. Based on our results, CH might be able to mitigate the negative effects that EOs can have on the dehydration of food products, improving the water barrier properties. Similarly to our observation, Ummarat and Seraypheap [52], studying the post-harvest effects of EOs on rambutan fruits (*Nephelium lappaceum* Linn.–Sapindaceae), found that *Cymbopogon nardus* L. (Poaceae) EO at concentrations higher than 0.04% enhanced the weight loss compared to their CTR. 

Another crucial attribute of meat products is the aesthetical quality in terms of surface colour. In our study, the application of plain CH or CH enriched with the EOs increased the lightness compared to the CTR beef: this could be an important feature for the consumers’ acceptability. The higher L* coordinate values at the beginning and after 7 days of storage were likely due to the coating itself. Indeed, Jo et al. [53] and Giatrakou et al. [54] found a similar effect in their studies regarding different CH treatments for meat preservation. Conversely, Lekjing [55] studied CH coatings with or without the addition of *Syzygium aromaticum* (L.) Merr. and Perry (Myrtaceae) EO applied on cooked pork sausages and found a decrease in this parameter when comparing treatments with CTR samples.

However, the coordinate a*, indicating redness, was similar to the CTR values until 4 days of storage, except for the CH enriched with the *L. nobilis* EO. At the end of the storage period, the lower a* induced by all treatments, except for the CH enriched with the *P. nigrum* EO, resulted in a less bright red colour, according to the instrument, which might suggest the likely ongoing oxidation processes. 

As the different redness could be caused by the CH coatings or EOs themselves, we also compared the time-course modifications for each treatment. That, indeed, revealed no significant changes in the patties coated with the EOs-enriched CH solutions, but a little time-dependent increase for the CTR and CH samples occurred. Myoglobin is the principal protein responsible for meat colour, and its oxygenation causes the conversion of this molecule into oxymyoglobin, which gives a bright red colour [56]. Then, over time, deoxy- and oxymyoglobin forms are further oxidised to metmyoglobin, causing the production of the brown colour of meat [57]. Therefore, it can be hypothesised that an oxygenation process of myoglobin in the CTR and CH samples was likely ongoing during the storage of beef patties, while the other treatments slowed down the oxygenation reactions. Even the increase at 4 days of storage of the L* coordinate of beef patties treated with the *P. nigrum* EO likely suggests changes in the protein structure caused by oxidation, as indicated by MacDougall [58]. Finally, the differences in total colour (ΔE) calculated for each group at both times of storage in respect of 0 days pointed out that untreated beef changed greatly and significantly compared to the EO-enriched and CH-treated samples. On the other hand, at each storage time, the higher ΔE_ab_ values were detected when chitosan was added to the meat, while the distance between the chromatic coordinates (∆E_ab_) showed how all the meat samples treated with different preserving solutions could not be distinguishably discriminated (∆E_ab_ < 6) in colour if compared with each other and control [59].

The results related to the TBARS concentration indicated that the lipid peroxidation status was affected by the treatments differently according to the different times of storage. Indeed, at 4 days, a positive influence of CH and the EOs alone was noticed compared to the untreated beef, confirming their ability to slow down the oxidative reactions occurring within the biological matrix. Similarly, Vital et al. [60] found a reduction in lipid peroxidation in beef treated with rosemary and oregano EOs compared to untreated beef. Moreover, CH is known for its antioxidant properties, as reported in other studies on beef [61,62]. 

After 7 days, the lipid peroxidation was similar to the CTR group for all treatments, meaning that the protective effect of the EOs noted at the previous time point faded. Indeed, the treatments with the EOs alone caused an increase in this lipid peroxidation index in comparison with the CH treatments. In particular, the *L. nobilis* EO, when added to the CH solution, performed better. This might indicate that, after 7 days, the EOs might have undergone a natural auto-oxidation of some lipid components that, instead, was prevented by the EO addition into the CH solution. This auto-oxidation was likely able to set off other oxidative reactions within the food matrix. Indeed, CH acts as a selective gas barrier, i.e., towards oxygen, as demonstrated by several other studies [63,64], the property might have protected the EOs included in it.

## 5. Conclusions

The results presented in this work show that edible coatings made of CH and selected EOs can be promising, innovative allies in beef meat protection. Regarding the smell profiles, the application of the *L. nobilis* or *P. nigrum* EOs, alone or mixed with CH, enhances the odour pleasantness of raw meat, masking the usual cadaverine-like smell. Interestingly, the *P. nigrum* EO enriched CH is significantly active in repelling the blowfly *C. vomitoria*, avoiding its oviposition on meat. That feature could be successfully exploited for the implementation of EOs-enriched CH sprayable coatings able to reduce meat loss and waste due to the Calliphoridae flies in slaughterhouses, industries, and stores where the hygienic conditions are not optimal. All the treatments proposed, compared to the control, do not accelerate meat dehydration and lipid peroxidation after 7 days of storage, preserving its organoleptic qualities and shelf-life. Interestingly, in earlier days, a pronounced antioxidant effect against lipid peroxidation was achieved with the EOs treatments, but this protection was transient and faded later. Furthermore, the treatments increase the colour lightness of meat, an attractive feature for consumers. 

## Figures and Tables

**Table 1:** Chemical compositions of the *Allium sativum*, *Laurus nobilis*, *Ocimum basilicum*, *Piper nigrum*, and *Salvia rosmarinus* essential oils (EOs).

Compound	l.r.i ^a^	Aroma Notes ^b^	Relative Abundance (%) ± SD ^c^				
			*A. sativum*	*L. nobilis*	*O. basilicum*	*P.nigrum*	*S. rosmarinus*
diallyl sulfide	866	sulphur	1.1 ± 0.28	- ^d^	-	-	-
(*Z*)-allyl(prop-1-en-1-yl)sulfane	888		0.5 ± 0.06	-	-	-	-
methyl allyl disulfide	916	garlic	1.1 ± 0.03	-	-	-	-
α-thujene	926		-	0.3 ± 0.01	-	0.2 ± 0.01	-
α-pinene	933		-	3.6 ± 0.03	0.2 ± 0.01	6.2 ± 0.01	9.3 ± 0.04
camphene	948	mint, fresh	-	0.3 ± 0.02	-	-	2.2 ± 0.03
1,2-dithiole	952	sulphur	0.5 ± 0.06	-	-	-	-
sabinene	973	wood	-	4.7 ± 0.02	-	4.2 ± 0.02	0.6 ± 0.07
dimethyl trisulfide	974	sulphur	0.4 ± 0.01	-	-	-	-
β-pinene	977		-	2.9 ± 0.02	0.2 ± 0.01	6.1 ± 0.05	5.9 ± 0.02
myrcene	991	wood	-	0.5 ± 0.00	0.1 ± 0.01	0.7 ± 0.01	0.8 ± 0.01
α-phellandrene	1006		-	0.2 ± 0.02	-	0.6 ± 0.03	0.3 ± 0.01
δ-3-carene	1011		-	0.1 ± 0.00	-	4.7 ± 0.03	0.2 ± 0.00
α-terpinene	1017		-	0.4 ± 0.00	-	-	0.4 ± 0.01
*p*-cymene	1025	lemon	-	0.5 ± 0.01	-	0.2 ± 0.01	3.1 ± 0.01
limonene	1029	lemon	-	1.2 ± 0.05	0.2 ± 0.00	8.0 ± 0.1	3.9 ± 0.03
1,8-cineole	1031	eucalyptus	-	28.1 ± 0.19	2.7 ± 0.02	-	41.1 ± 0.18
(*E*)-β-ocimene	1047		-	-	0.6 ± 0.01	-	-
γ-terpinene	1058		-	0.6 ± 0.01	-	-	1.0 ± 0.00
diallyl disulfide	1082	sulphur	6.7 ± 0.13	-	-	-	-
fenchone	1089		-	-	0.1 ± 0.01	-	-
terpinolene	1089		-	0.2 ± 0.00	-	0.3 ± 0.01	0.3 ± 0.01
linalool	1101	citrus	-	5.5 ± 0.11	0.6 ± 0.01	0.3 ± 0.01	0.3 ± 0.01
(*E*)-1-allyl-2-(prop-1-en-1-yl) disulfane	1103		0.3 ± 0.02	-	-	-	-
(*Z*)-1-allyl-2-(prop-1-en-1-yl) disulfane	1107		0.9 ± 0.09	-	-	-	-
fenchol	1114		-	-	0.1 ± 0.01	-	-
methyl allyl trisulphide	1142		5.4 ± 0.05	-	-	-	-
camphor	1145	camphor	-	-	0.4 ± 0.01	-	14.3 ± 0.11
4-methyl-1,2,3-trithiolane	1154		5.0 ± 0.22	-	-	-	-
borneol	1165		-	0.1 ± 0.01	-	-	2.7 ± 0.06
δ-terpineol	1166		-	0.3 ± 0.03	-	-	-
menthol	1173	mint	-	-	0.3 ± 0.00	-	-
4-terpineol	1177	spicy wood	-	2.1 ± 0.01	0.3 ± 0.02	0.3 ± 0.00	0.6 ± 0.02
cryptone	1186		-	-	-	-	0.1 ± 0.00
α-terpineol	1191		-	1.7 ± 0.01	-	-	1.3 ± 0.03
methyl chavicol	1196	sweet, phenolic	-	-	76.3 ± 0.50	-	-
fenchyl acetate	1221	sweet, balsamic	-	-	0.3 ± 0.00	-	-
*trans*-ascaridol glycol	1268		-	-	-	-	0.2 ± 0.00
linalyl acetate	1257	bergamot	-	0.3 ± 0.02	-	-	-
4-thujen-2-α-yl acetate	1273		-	0.1 ± 0.00	-	-	-
bornyl acetate	1286	menthol	-	0.6 ± 0.00	0.3 ± 0.01	-	1.7 ± 0.01
2-undecanone	1294	cheesy cream	-	0.1 ± 0.00	-	-	-
*di*-2-propenyl trisulfide	1297	garlic	18.3 ± 0.51	-	-	-	-
δ-terpinyl acetate	1315		-	0.8 ± 0.00	-	-	-
(*Z*)-1-allyl-3-(prop-1-en-1-yl)trisulfane	1327		5.0 ± 0.23	-	-	-	-
δ-elemene	1338		-	-	-	2.1 ± 0.01	-
α-terpinyl acetate	1350		-	17.5 ± 0.24	-	-	-
α-cubebene	1350		-		-	0.2 ± 0.00	-
eugenol	1357	sweet wood	-	3.4 ± 0.15	-	-	-
5-methyl-1,2,3,4-tetrathiane	1364		5.9 ± 0.37	-	-	-	-
neryl acetate	1365	floral	-	0.1 ± 0.00	-	-	-
α-ylangene	1371		-	0.1 ± 0.01	-	-	-
cyclosativene	1371		-	-	-	0.1 ± 0.01	-
α-copaene	1376		-	-	-	2.7 ± 0.02	0.3 ± 0.01
β-cubebene	1390		-	-	-	0.2 ± 0.00	-
β-elemene	1392		-	1.0 ± 0.02	0.3 ± 0.00	1.2 ± 0.03	-
methyl eugenol	1405	cinnamon	-	7.3 ± 0.08	0.8 ± 0.00	-	-
*iso*caryophyllene	1407	wood	-	-	-	0.1 ± 0.01	-
α-gurjunene	1410		-	0.1 ± 0.00	-	0.2 ± 0.01	-
β-caryophyllene	1419		-	1.7 ± 0.02	0.3 ± 0.01	45.7 ± 0.18	6.9 ± 0.04
1-(1-(methylthio)propyl)-2-propyldisulfane	1431		0.4 ± 0.00	-	-	-	-
*trans-*α-bergamotene	1436		-	-	5.4 ± 0.06	-	-
α-guaiene	1439		-	0.2 ± 0.01	0.2 ± 0.03	0.4 ± 0.01	-
(*E*)-cinnamyl acetate	1444		-	0.1 ± 0.03	-	-	-
α-humulene	1453		-	0.2 ± 0.01	0.1 ± 0.00	3.4 ± 0.02	0.8 ± 0.01
*allo*aromadendrene	1460	wood	-	0.2 ± 0.00	-	-	-
*cis*-muurola-4(14),5-diene	1463		-	-	0.1 ± 0.00	-	-
γ-muurolene	1477		-	-	-	0.1 ± 0.00	0.3 ± 0.01
germacrene D	1481		-	0.2 ± 0.00	-	2.0 ± 0.02	-
β-selinene	1486		-	0.3 ± 0.01	-	2.2 ± 0.02	-
valencene	1493		-	-	-	-	0.1 ± 0.00
α-selinene	1495		-	0.2 ± 0.06	-	1.7 ± 0.05	-
bicyclogermacrene	1496	green wood	-	0.8 ± 0.05	-	0.2 ± 0.01	-
*iso*methyleugenol	1497		-	0.1 ± 0.00	-	-	-
α-muurolene	1500		-	-	-	0.3 ± 0.02	-
α-bulnesene	1505		-	0.2 ± 0.01	0.2 ± 0.01	-	-
β-bisabolene	1509		-	-	-	0.2 ± 0.01	-
*trans*-γ-cadinene	1514		-	0.4 ± 0.00	1.4 ± 0.04	-	0.2 ± 0.01
β-sesquiphellandrene	1524		-	-	-	-	-
δ-cadinene	1524		-	0.9 ± 0.01	0.2 ± 0.01	1.2 ± 0.01	0.6 ± 0.00
diallyl tetrasulphide	1538	garlic	27.3 ± 0.47	-	-	-	-
*cis*-sesquisabinene hydrate	1543		-	0.3 ± 0.01	-	-	-
germacrene B	1556	wood	-	-	-	0.2 ± 0.01	-
elemicin	1558	floral	-	0.5 ± 0.01	-	-	-
*p*-methoxycinnamaldehyde	1567	cherry, vanilla	-	-	2.3 ± 0.15	-	-
*trans*-*p*-methoxycinnamaldehyde	1569		-	-	1.3 ± 0.04	-	-
spathulenol	1577	herbaceous	-	1.4 ± 0.05	0.2 ± 0.01	-	-
caryophyllene oxide	1582		-	1.9 ± 0.08	0.2 ± 0.00	2.3 ± 0.07	0.5 ± 0.01
1-(1-(prop-1-en-1-ylthio)propyl)-2-propyl disulfane	1592		0.2 ± 0.00	-	-	-	-
viridiflorol	1592		-	0.4 ± 0.02	-	-	-
6-methyl-4,5,8-trithia-1,10-undecadiene	1598		0.6 ± 0.01	-	-	-	-
humulene oxide II	1608		-	0.2 ± 0.02	-	0.1 ± 0.00	-
1,10-*di*-*epi*-cubenol	1615		-	-	0.4 ± 0.00	-	-
1-*epi*-cubenol	1627		-	0.4 ± 0.04	-	0.8 ± 0.05	-
γ-eudesmol	1631		-	0.2 ± 0.04	-	-	-
caryophylla-4(14),8(15)-dien-5-ol	1633		-	0.2 ± 0.03	-	-	-
*Iso*sphatulenol	1640		-	0.2 ± 0.07	-	-	-
τ-cadinol	1641		-	0.6 ± 0.08	3.5 ± 0.13	-	-
δ-cadinol	1645		-	0.2 ± 0.05	-	-	-
τ-muurolol	1646		-	-	-	0.2 ± 0.01	-
β-eudesmol	1649		-	0.7 ± 0.09	-	-	-
α-eudesmol	1653		-	0.5 ± 0.02	-	-	-
α-cadinol	1654		--	0.6 ± 0.01	-	0.1 ± 0.01	-
pogostole	1655		-	0.2 ± 0.00	-	-	-
aromadendrene epoxide II	1680		-	0.2 ± 0.01	-	-	-
eudesm-4(15),7-dien-1β-ol	1686		-	0.1 ± 0.01	-	-	-
1-allyl-3-(2-(allylthio)propyl)trisulfane	1818		5.4 ± 0.20	-	-	-	-
*m*-camphorene	1952	kaempferol	-	-	-	0.3 ± 0.02	-
*p*-camphorene	1986		-	-	-	0.1 ± 0.01	-
1-allyl-3-(2-(allyldisulfanyl)propyl)trisulfane	2066		1.1 ± 0.01	-	-	-	-
Monoterpene hydrocarbons			-	15.6 ± 0.15	1.2 ± 0.03	31.3 ± 0.09	28.1 ± 0.10
Oxygenated monoterpenes			-	57.2 ± 0.54	5.1 ± 0.07	0.5 ± 0.01	62.0 ± 0.03
Sesquiterpenes hydrocarbons			-	6.4 ± 0.04	8.3 ± 0.15	64.2 ± 0.06	9.3 ± 0.06
Oxygenated sesquiterpenes			-	8.3 ± 0.58	4.3 ± 0.15	3.5 ± 0.14	0.5 ± 0.01
Phenylpropanoids			-	11.4 ± 0.08	80.8 ± 0.39	-	-
Diterpenes hydrocarbons			-	-	-	0.4 ± 0.03	-
Other non-terpene derivatives			-	0.3 ± 0.03	-	-	0.1 ± 0.00
Sulphur derivatives			86.1 ± 0.08	-	-	-	-
Total identified (%)			86.1 ± 0.08	99.2 ± 0.03	99.6 ± 0.01	100.0 ± 0.00	100.0 ± 0.00

^a^ Linear retention index on a HP-5MS capillary column; ^b^ aroma notes from TGSC [29]; ^c^ standard deviation; ^d^ not detected.

**Table 2:** Main odours that characterised the smell of the *Allium sativum*, *Laurus nobilis*, *Ocimum basilicum*, *Piper nigrum*, and *Salvia rosmarinus* essential oils (EOs).

Odorant Notes	*A. sativum*	*L. nobilis*	*O. basilicum*	*P. nigrum*	*S. rosmarinus*
Fruity		Fresh fruits		Citrus	
				Mandarin	
				Grapefruit	
Floral				Lilac	Dried flowers
				Wisteria	
				Orange blossom	
Vegetal		Fresh vegetables	Anise	Eucalyptus	Eucalyptus
			Mint		Mint
					Mentholated
Spicy		Resin	Sandalwood		
		Sandalwood	Liquorice		
Off-flavours	Burnt garlic				Methane
	Emetic				
	Sulphur				

**Table 3:** Colour coordinates (L*, a*, b*) of beef meat samples in cubic embedding moulds. In each column, different letters (a–g) indicate statistically significant differences.

Sample	L*	a*	b*
Meat	43.34 ± 0.09 ^g^	14.62 ± 0.07 ^c^	1.15 ± 0.34 ^de^
Meat + CH	46.29 ± 0.04 ^f^	17.69 ± 0.02 ^a^	1.66 ± 0.01 ^d^
Meat + CH + *A. sativum* EO (up)	47.44 ± 0.01 ^c^	5.33 ± 0.01 ^f^	10.56 ± 0.03 ^a^
Meat + CH + *A. sativum* EO (down)	47.15 ± 0.01 ^d^	11.60 ± 0.01 ^e^	3.32 ± 0.01 ^c^
Meat + CH + *L. nobilis* EO	47.38 ± 0.08 ^cd^	14.92 ± 0.04 ^b^	0.07 ± 0.04 ^f^
Meat + CH + *O. basilicum* EO	46.65 ± 0.13 ^e^	13.27 ± 0.10 ^d^	3.49 ± 0.30 ^c^
Meat + CH + *P. nigrum* EO	50.92 ± 0.02 ^a^	11.45 ± 0.01 ^e^	0.63 ± 0.02 ^ef^
Meat + CH + *S. rosmarinus* EO	49.74 ± 0.01 ^b^	11.43 ± 0.01 ^e^	5.59 ± 0.01 ^b^

Chitosan (CH); *Allium sativum*, *Laurus nobilis*, *Ocimum basilicum*, *Piper nigrum*, or *Salvia rosmarinus* essential oil (EO); EOs-enriched CH solutions (e.g., CH + A. sativum EO).

**Table 4:** L*, a*, and b* parameters (CIELAB) of beef patties subjected to different treatments for 7 days of cold storage.

Treatments	Coordinate	Time of Storage		
		0 Days	4 Days	7 Days
Meat	L*	38.84 ± 1.80 ^C^	41.36 ± 2.25	39.78 ± 2.30 ^B^
	a*	19.63 ± 1.11 ^b^	22.458 ± 1.46 ^a,A^	23.70 ± 1.88 ^a,A^
	b*	5.328 ± 0.95 ^AB^	5.02 ± 0.80	6.16 ± 1.05
	∆E_ab_*		4.03	4.20
Meat + CH	L*	42.91 ± 1.49 ^A^	43.07 ± 1.07	43.46 ± 1.03 ^A^
	a*	18.86 ± 1.92 ^b^	21.34 ± 1.37 ^a,AB^	21.28 ± 0.82 ^a,B^
	b*	4.12 ± 1.24 ^B^	5.13 ± 0.74	5.24 ± 0.94
	∆E_ab_*		2.88	3.12
Meat + CH + *L. nobilis* EO	L*	42.97 ± 1.83 ^A^	44.24 ± 1.80	43.99 ± 1.86 ^A^
	a*	20.01 ± 1.08	19.92 ± 0.98 ^B^	20.51 ± 0.46 ^B^
	b*	5.01 ± 0.55 ^AB^	4.65 ± 1.12	5.51 ± 0.54
	∆E_ab_*		2.07	1.89
Meat + CH +*P. nigrum* EO	L*	42.57 ± 1.71 ^AB^	43.52 ± 1.80	43.38 ± 1.36 ^A^
	a*	21.26 ± 1.32	21.86 ± 1.31 ^AB^	21.81 ± 1.32 ^AB^
	b*	6.17 ± 1.28^A^	5.79 ± 1.00	6.41 ± 0.98
	∆E_ab_*		2.18	1.21
Meat + *L. nobilis* EO	L*	40.65 ± 2.77 ^ABC^	41.88 ± 2.56	40.37 ± 1.65 ^B^
	a*	19.72 ± 1.36	20.45 ± 1.62 ^AB^	20.94 ± 1.52 ^B^
	b*	5.87 ± 0.55 ^A^	5.53 ± 0.93	5.91 ± 0.10
	∆E_ab_*		1.65	2.98
Meat + *P. nigrum* EO	L*	39.41 ± 1.67 ^b,BC^	41.77 ± 1.24 ^a^	41.17 ± 1.41 ^ab,AB^
	a*	20.50 ± 1.20	20.57 ± 1.46 ^AB^	20.97 ± 1.63 ^B^
	b*	6.31 ± 0.67 ^A^	5.82 ±0.66	6.58 ± 0.62
	∆E_ab_*		2.63	2.39

For each parameter, different letters indicate significant differences according to Tukey HSD (*p* ≤ 0.05). Data are expressed as mean ± standard deviation. Upper case letters (A–C) were used to indicate differences according to the meat treatment within each day of storage; lower case letters (a–b) were used to indicate differences according to the time of storage for each meat treatment. Chitosan (CH), *Laurus nobilis* or *Piper nigrum* essential oil (EO), and EOs enriched CH solutions (CH + *L. nobilis* EO; CH + *P. nigrum* EO).

**Table 5:** CIE L*a*b* color differences (∆E^∗^_ab_) among samples.

(a) 0 Days of Storage	Meat	Meat + CH	Meat + CH + *L. nobilis* EO	Meat + CH + *P. nigrum* EO	Meat + *L. nobilis* EO	Meat + *P. nigrum* EO
Meat		4.32	4.16	4.16	1.89	1.43
Meat + CH			1.16	3.17	2.99	4.44
Meat + CH + *L. nobilis* EO				1.75	2.44	3.82
Meat + CH + *P. nigrum* EO					2.48	3.25
Meat + *L. nobilis* EO						1.53
**(b)** **4 days of storage**	**Meat**	**Meat + CH**	**Meat + CH + *L. nobilis* EO**	**Meat + CH + *P. nigrum* EO**	**Meat + *L. nobilis* EO**	**Meat + *P. nigrum* EO**
Meat		2.04	3.86	2.37	2.14	2.09
Meat + CH			1.9	0.95	1.54	1.66
Meat + CH + *L. nobilis* EO				2.36	2.57	2.81
Meat + CH + *P. nigrum* EO					2.17	2.17
Meat + *L. nobilis* EO						0.34
**(c)** **7 days of storage**	**Meat**	**Meat + CH**	**Meat + CH + *L. nobilis* EO**	**Meat + CH + *P. nigrum* EO**	**Meat + *L. nobilis* EO**	**Meat + *P. nigrum* EO**
Meat		4.49	5.32	4.07	2.83	3.1
Meat + CH			0.97	1.27	3.67	2.67
Meat + CH + *L. nobilis* EO				1.69	3.17	3.04
Meat + CH + *P. nigrum* EO					3.17	2.37
Meat + *L. nobilis* EO						1.04

## Data Availability

The datasets are available on request from the corresponding author.
